# Shaping an Effective Health Information Website on Rare Diseases Using a Group Decision-Making Tool: Inclusion of the Perspectives of Patients, Their Family Members, and Physicians

**DOI:** 10.2196/ijmr.7352

**Published:** 2017-11-20

**Authors:** Ana Babac, Svenja Litzkendorf, Katharina Schmidt, Frédéric Pauer, Kathrin Damm, Martin Frank, Johann-Matthias Graf von der Schulenburg

**Affiliations:** ^1^ Center for Health Economic Research Hannover University Hannover Hannover Germany; ^2^ Center for Quality and Management in Health Care Medical Association of Lower Saxony Hannover Germany

**Keywords:** rare diseases, decision making, health information needs, preferences, patients, relatives, physicians

## Abstract

**Background:**

Despite diverging definitions on rare conditions, people suffering from rare diseases share similar difficulties. A lack of experience by health professionals, a long wait from first symptoms to diagnosis, scarce medical and scientific knowledge, and unsatisfactory treatment options all trigger the search for health information by patients, family members, and physicians. Examining and systematically integrating stakeholder needs can help design information platforms that effectively support this search.

**Objective:**

The aim of this study was to innovate on the group decision-making process involving patients, family members, and physicians for the establishment of a national rare disease Internet platform. We determined differences in the relevance of health information—especially examining quantifiable preference weights—between these subgroups and elucidated the structure and distribution of these differences in people suffering from rare diseases, their family members, and physicians, thus providing information crucial to their collaboration.

**Methods:**

The included items were identified using a systematic Internet research and verified through a qualitative interview study. The identified major information needs included *medical issues*, *research*, *social help offers*, and *current events*. These categories further comprised sublevels of *diagnosis*, *therapy*, *general disease pattern*, *current studies*, *study results*, *registers*, *psychosocial counseling*, *self-help*, and *sociolegal advice*. The analytic hierarchy process was selected as the group decision-making tool. A sensitivity analysis was used to determine the stability and distribution of results. *t* tests were utilized to examine the results’ significance.

**Results:**

A total of 176 questionnaires were collected; we excluded some questionnaires in line with our chosen consistency level of 0.2. Ultimately, 120 patients, 24 family members, and 32 physicians participated in the study (48 men and 128 women, mean age=48 years, age range=17-87 years). Rankings and preference weights were highly heterogeneous. Global ranking positions of patients, family members, and physicians are shown in parentheses, as follows: *medical issues* (3/4, 4, 4), *research* (3/4, 2/3, 3), *social help offers* (1, 2/3, 2), and *current events* (2, 1, 1); *diagnosis* (6, 8, 9), *therapy* (5, 9, 7), *general disease pattern* (9, 4/5/6, 6), *current studies* (7, 4/5/6, 3), *study results* (8, 7, 8), *registers* (4, 1, 5), *psychosocial counseling* (1, 2, 4), *self-help* (3, 3, 2), and *sociolegal advice* (2, 4/5/6, 1). Differences were verified for patients for 5 information categories *(P*=.03), physicians for 6 information categories (*P*=.03), and family members for 4 information categories (*P*=.04).

**Conclusions:**

Our results offer a clear-cut information structure that can transparently translate group decisions into practice. Furthermore, we found different preference structures for rare disease information among patients, family members, and physicians. Some websites already address differences in comprehension between those subgroups. Similar to pharmaceutical companies, health information providers on rare diseases should also acknowledge different information needs to improve the accessibility of information.

## Introduction

Worldwide, approximately 350 million people are affected by rare disease [1]. Despite diverging definitions, people suffering from rare diseases share common difficulties. Particularly, health care professionals have little experience with this patient group, and patients typically wait a long time from the first symptoms to diagnosis. Moreover, medical and scientific knowledge concerning rare diseases is scarce, and low research efforts often result in, if available, unsatisfactory treatment options. When there is a treatment option available, patients still often need to consider financial aspects. Patients also frequently experience difficulties with the cost absorption of expensive treatments. Furthermore, rare diseases are very serious and chronic. Severe symptoms result in high disease burden and can have a significant negative impact on one’s quality of life. Above all, patients often face a shortened life expectancy [2]. Consequently, there is an urgent need for proper health information for this population.

The Internet offers a large pool of somewhat obscure information. In this context, this study examines how information on rare diseases can be presented in a more structured way. As a second step, we also examined whether stakeholder-specific websites presenting information in accordance with the information priorities of the targeted subgroups would be necessary. We hypothesized that the information structures of patients, family members, and physicians would be identical, as family members and physicians would generally search for information to fulfill patients’ needs. This would consequently lead to a single platform incorporating the overall group consensus on information priorities and therefore information presentation.

The literature, however, has not yet addressed the differing information needs between patients, family members, and physicians. Health information helps to empower patients, enabling them to understand, treat, cope, and effectively manage their disease [3-5]. Rare diseases’ patients are often called *experts* of their own illnesses because they gather health information consciously through Web searches or unconsciously through numerous consultations with different health care professionals [6]. Besides, doctors’ assessments of patients’ preferences appear to be critical for the outcome of health services [7]. In this regard, the dialogue between patients and physicians is critical. Therefore, health care professionals must be trained and prepared to listen to patients and discuss their experiences [8,9]. Furthermore, health information searches should be facilitated and encouraged, as they enable patients to be more effective in communicating with their physicians [5]. This study contributes and adds value to this existing literature and the underlying dialogue by eliciting the different perspectives of patients, family members, and physicians on the relevance of rare disease information.

Aside from the above points, little or no scientific knowledge exists for the 5000 to 6000 different indications summarized under the term rare diseases. Adding all diseases and all different information providers together creates a huge and obscure information pool. Indeed, information providers often fail to meet the information needs of patients and families searching social media and utilizing chat rooms to obtain information; however, they might be unaware of the low quality of this information [10]. On the other hand, obtaining knowledge of the many thousands of different rare diseases is well beyond the ability of physicians. Primary physicians are only familiar with approximately 400 different indications. Primary physicians can extend their knowledge through asking questions of colleagues and reviewing paper-based data sources [11]; however, even with the advent of electronic records, it remains highly time-consuming and difficult to search for the right terms and obtain appropriate evidence. Taken together, these facts suggest that effective health information presentation is exceedingly important. Collins et al suggest that information needs can be incorporated by capturing and embedding the relevance of information [12]. This study shows how this demand can be put into practice.

Literature shows that group decision-making tools are rarely applied when it comes to the establishment of health information portals. Health information needs are often met by retrieving information from historic user statistics or triggering retrospection. Stakeholders cannot actively participate [13,14]. However, by choosing the analytic hierarchy process as a group decision-making tool, we can actively involve patients, family members, and physicians to address their unmet informational needs. Furthermore, information categories that are underrated by stakeholders (ie, patients, relatives, or physicians) can be illuminated. A number of different models have already been applied during the establishment of effective cocreative business modeling [15,16]. However, until now, there have been no attempts to devise a similar model in a transparent manner for different stakeholders in relation to rare diseases.

The following study has been conducted against the backdrop of the conceptualization of a central website for rare disease information in Germany (ZIPSE, Zentrales Informationsportal über seltene Erkrankungen or central information portal about rare diseases) [17] connecting disease unspecific and specific information, as well as quality orientation for patients, their families, and health care professionals at a central platform [18]. As part of the German National Action Plan for Rare Diseases from 2013 (NAMSE, Nationales Aktionsbündnis für Seltene Erkrankungen) following the European council recommendations [19,20], knowledge transfer is improved through the development of Internet information systems. Already existing Internet information is collected and organized to increase the visibility of rare disease knowledge [18]. Physicians, family members, and patients are critical to this process; they are the major beneficiaries and should profit by effective health information provision.

In this paper, we describe how patients, family members, and physicians can contribute directly to this process of effectively gathering and presenting health information. More specifically, we describe an innovative group decision-making process involving these individuals aimed at establishing a national rare diseases Internet platform. This study also examined the information preferences of these stakeholders to enable health care systems, decision makers, and other national and international rare diseases portals to appropriately structure information that patients, families, and physicians strive for. The relevance of information is crucial for stakeholders’ ability to relate to each other within a strong network approach. In this regard, the study provides unique insights into the quantitative structure and distribution of information preferences for these stakeholders, answering the question on how information provision in the context of rare diseases should be structured.

## Methods

### Ethical Considerations

The questionnaire was distributed both Web-based and as a paper-based version. Accordingly, consent was obtained in written form. The paper-based version was distributed after qualitative interviews with patients and their relatives. A positive ethics committee vote was obtained for the interview study from the ethics committee at Albert Ludwigs University of Freiburg (number 53/14). The Web-based version allowed for collecting opinions anonymously without having participants disclose personal details at any time. An information sheet was presented to all participants describing the aim and scope of the study. All participants were informed that they could withdraw from the study at any time.

### Analytic Hierarchy Process (AHP)

An analytic hierarchy process (AHP) was implemented for the collection of individual preferences, as this study was devised to contribute the decision-making processes implemented in the ZIPSE project. Saaty gives detailed information on the AHP methodology [21]. Two authors also give a detailed overview of its application in health care [22,23]. Lately, the Institute for Quality and Efficiency in Health Care in Germany discussed the AHP as a method for the inclusion of preference structures into early benefit assessment. Similar to conjoint analysis, AHP raises quantifiable weights that can then be used to combine multiple endpoints into an efficiency boundary [24,25]. AHP offers a direct approach, whereas conjoint analysis compares different attributes in combination, thereby leading to an indirect calculation of weights. Furthermore, it is more intuitive and easier to understand for inexperienced participants compared with other techniques (eg, the analytic network process [26] but more informative than other techniques, eg, best-worst scaling, ranking) [27]). Quantitative preference distances make extensive evaluation of preference structures possible [20,28]. Therefore, the major benefit to AHP methodology is that it raises not only ranks but also measurable distances between criteria weights, leading to a visible preference structure. AHP does not only give a clear-cut ranking, it also indicates what categories are weighted similarly. Therefore, attributes that are weighted similarly, but ranked differently, do not need to be excluded. The AHP is able to appreciate individual judgments adequately to thereby derive an overall group consensus [29] and offers a clear-cut preference structure that can be easily applied to the presentation of health information.

AHP is particularly interesting for the field of rare diseases as it is applicable independent of the size of the indication. Even opinions of very small rare disease subgroups can be raised and evaluated [20,28]. Moreover, AHP appreciates the heterogeneity of rare diseases, which because of its definition, summarizes quite diverging disease patterns, as subgroup specific opinions can be evaluated separately. Consequently, this study recognizes the value of AHP when examining rare diseases.

### Hierarchy Definition

A total of 300 information websites addressing rare diseases were searched and scanned concerning available information on their home pages. Litzkendorf et al also collected and verified the items through a qualitative interview study [30]. Similar information categories have also been found by the Genetic and Rare Disease Information Center [31] and for other indications such as multiple sclerosis [32]. Accordingly, information categories were drafted and prestructured. Four experts in public health research and one expert in health economics research were chosen from the Center for Health Economics Research Hannover (CHERH). The major criterion for choosing these experts was a research focus on either rare diseases or patient-reported outcomes. Participants were addressed personally. An invitation for participation was forwarded via email along with an attached Microsoft Excel 2010 sheet containing the included items. Afterwards, the final definition of the items was discussed in a workshop scenario. As a result, the different information category descriptions address biases because of different interpretations of information categories. Definitions were finalized if they seemed closed to interpretation and easily understandable (see Multimedia Appendix 1). Thirteen items were chosen, which resulted in 15 pairwise comparisons. The final hierarchy is presented in Table 1.

### Questionnaire Development

Other studies used computer-based programs that immediately reflected the level of consistency generated by the answer [33]. Then, corrections are initiated. However, in our study, we did not use an intelligent computer-based fill-out system, instead implemented a paper-based questionnaire. A first draft of the questionnaire was designed and pretested. The pretest revealed insufficient consistency. Therefore, the questionnaire was redrafted. A graphic showing the hierarchy structure was removed to allow space for a graphic demonstrating the exemplary filling out of one question on the questionnaire. Furthermore, a ranking task was integrated, which visualized the intrinsic priorities during the fill-out process. A research question was specified for each visual scale.

**Table 1:** Hierarchy for information on rare diseases.

Hierarchy level 1	Hierarchy level 2	Hierarchy level 3
Research topic	Parameters	Elements
Importance of health information on rare diseases	Medical issues	Diagnosis
		Therapy
		General disease pattern
	Research	Current studies
		Study results
		Registers
	Social help offers	Psychosocial counseling
		Self-help counseling
		Sociolegal advice
	Current events	

The end of a paragraph containing items from one hierarchy arm was highlighted to emphasize the beginning of a new category. A subsequent pretest revealed improved consistency. Before fielding the questionnaire, the usability and technical functionality of its Web-based version were tested by the authors and a collaborating institution (see Multimedia Appendix 2).

### Sample

Patients, physicians, and family members were identified as the main users of health information on rare diseases [34] and a central rare diseases information portal [20]. Participants were recruited using three different recruiting strategies to ensure the adequacy of the sample. The Freiburg Center for Rare Diseases located at the Department of Dermatology of the University Medical Center, University of Freiburg contacted patients and family members using rare diseases self-help groups. Overall, 39 individuals were asked to complete the questionnaire. To participate in the study, patients had to be aged 17 years and older; if they were younger than 18 years, a close relative was invited for answering the questions instead. Interviews were predominately conducted via telephone. To ensure a broad and balanced representation of patients suffering from rare diseases, eleven groups of rare diseases were formed when this study commenced; this was believed to represent considerable variety in rare diseases. Patients were recruited in accordance with these groups. Physicians were recruited by the CHERH. First, physicians with experience in rare diseases and working for specialized rare diseases centers were recruited. Later, the target group was extended to include physicians not imperatively familiar with rare diseases. This seems legitimate, as opinions of physicians unfamiliar with rare diseases but also searching for information were included. Furthermore, a Web-based version of the questionnaire was devised. The link to the open Web-based version was stored on a website offering Web surveys and forwarded by Alliance for chronic rare diseases (Allianz chronisch seltener Erkrankungen, ACHSE) using a mailing list of ACHSE members. A short description of the study was included. All data were collected and stored anonymously. ACHSE checked the avoidance of identification of rare diseases’ patients through disease characteristics. The study was initiated in August 2014, and data collection was finalized in August 2016. Overall, 112 questionnaires were answered online, and 64 paper-based questionnaires were completed.

### Analysis

For each respondent, a consistency ratio (CR) was calculated. The CR was calculated in accordance with the following formula: (λ_max−_ n)/(n−1). λ_max_. The CR is a value which has been predefined by Saaty [21]. Following the threshold of Danner et al, we included all comparisons with a CR≤0.2; therefore, we assumed pairwise comparisons to be consistent up to this threshold [35]. Respondents with a higher CR were excluded. Individual priority vectors were calculated using the eigenvector method used in Saaty [21]. Afterwards, individual opinions were summarized using an aggregation of individual priorities method. As literature suggests that values must correspond to reciprocal values of individual participants, weights were aggregated choosing the geometric means calculation [27]. As priority values need to sum up to one, resulting local priorities were weighted accordingly. Then, local and global rankings were derived. The calculation was conducted using Microsoft Excel 2010 and R version 3.1.2 (R-project for statistical computing). Responses of patients, families, and physicians were compared. To compare differences between these three subgroups, a variance analysis should be conducted first. However, as we analyzed differences between each of the three groups, test statistics were calculated using a student *t* test. Only local weights were compared as global weights were derived from these. An analysis of sensitivity was conducted observing the stability of priority rankings. Typically, AHP studies conduct sensitivity analysis using expert choice and graphically altering the weights of decision criteria and observing how rankings of alternatives outcomes change. However, this study did not include a hierarchy level with alternative decision outcomes, only items. Therefore, we assessed the sensitivity by identifying outliers and excluding them. Thereafter, potential rank reversals were observed. The range of data was elicited by box plots.

Bootstrapping (N=1000) was conducted to assess the proximity of values in correspondence to the parameter of the population, especially acknowledging small samples in the groups of family members and physicians.

## Results

### Sample Characteristics

The mean CR was 0.22 (median: 0.14, standard deviation, SD=0.24) for all 176 participants. Questionnaires with a CR above 0.2 were excluded. A mean CR was calculated for each subgroup. CR for all people suffering from a rare disease was 0.25 (SD=0.27), CR for families was 0.17 (SD=0.11), and CR for physicians was 0.14 (SD=0.10). Accordingly, the proportion of consistent answers was 56% for patients, 67% for relatives, and 83% for physicians, showing that most of the inconsistencies occurred in the patient subgroup. Solely regarding consistent answers, average CR for all participants was 0.09 (SD=0.05). Characteristics of all participants are shown in Table 2, including participants who answered inconsistently. Physicians were not asked about their civil status or the number of household members because this did not seem to serve our research question. Furthermore, disease severity and age of diagnosis were not applicable for two subgroup.

### Information Priorities

Tables 3-5 show both global and local priorities of level 2 and 3 items for all participants interviewed. Standard deviations of local priority weights are presented. Resulting ranks are also listed. As bootstrapping showed that calculated geometric means systematically underestimated the weights of information category, weighted geometric means were calculated. Results are presented separately for each subgroup.

### Sensitivity Analysis

**Figure 1:**
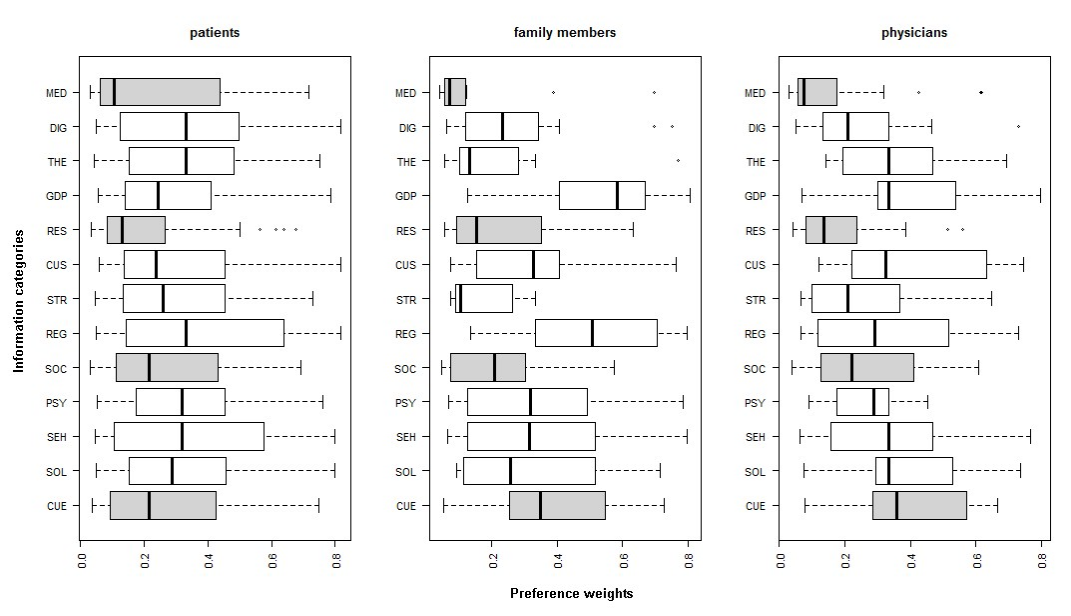
Range of results (local weights) of consistent answers by patients, family members, and physicians. CUS: current studies; DIG: diagnosis; GDP: general disease pattern; MED: medical issues; THE: therapy; PSY: psychosocial counseling; REG: registers; RES: research; SOC: social help offers; SHE: self-help; SOL: sociolegal advice; STR: study results.

The results range is displayed in Figure 1 and shows the potential sensibility of local weights to outliers. The ranking results were calculated based on the geometric means because the literature suggests that this procedure is more precise [27]. However, the following box plots show the range of results in a more intuitive manner, displaying the average mean, as well as the maximum and minimum local weights.

To test for potential rank reversal, we excluded outliers and observed whether rank reversals were of consequence. Figure 1 identifies the outliers visually. The patient subgroup displays only one outlier that results in a rank reversal for the category *research*. *Research* is consequently ranked last with a priority weight of .19. Family members show outliers for categories *medical information* (.09), *therapy* (.21), *diagnosis* (.19), and *general disease pattern* (.60). The exclusion of outliers does not cause rank reversal. For the last group, *physicians*, outliers were identified for the following items: *medical information* (.11), *diagnosis* (.22), and *research* (.17). No rank reversals were observed.

### Significance of Results

To examine differences between groups, we conducted a student *t* test, assuming opinions were aggregated following the normal distribution within the population. The results are displayed in Table 6. The null hypothesis states that the importance of items is perceived equally; the alternative hypothesis states that the importance of information on rare diseases is perceived differently. Significant differences are marked.

**Figure 2:**
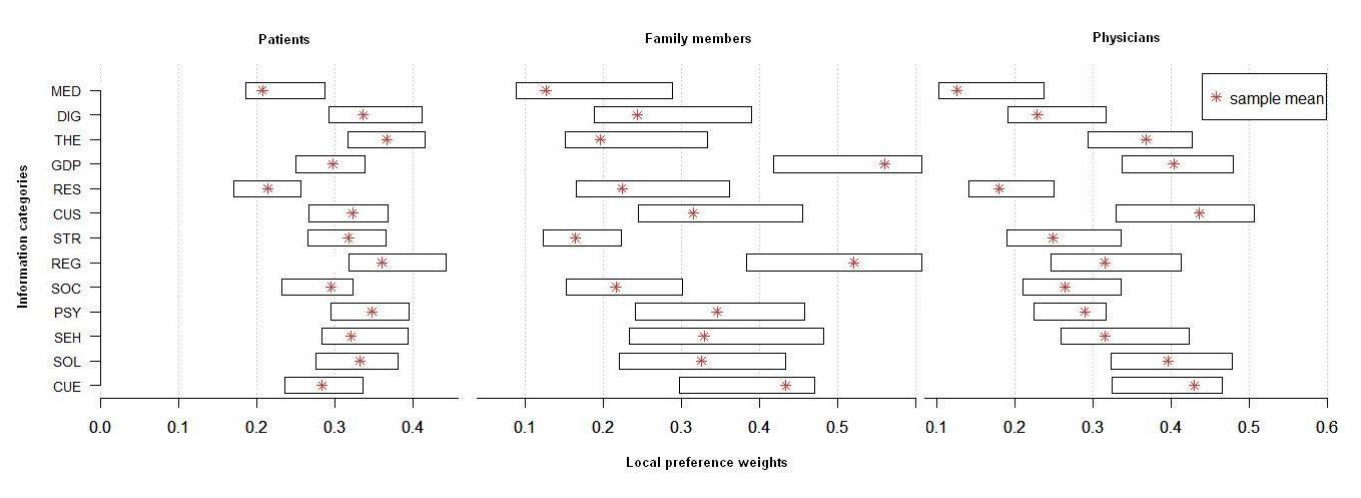
The results of patients, family members, and physicians using bootstrapping and a 95% CI. CUS: current studies; DIG: diagnosis; GDP: general disease pattern; MED: medical issues; THE: therapy; PSY: psychosocial counseling; REG: registers; RES: research; SOC: social help offers; SHE: self-help; SOL: sociolegal advice; STR: study results.

Furthermore, bootstrapping with a 95% CI was conducted to examine whether sample results lay within specific ranges of the population regarded. The results are presented in Figure 2.

**Table 2:** Sociodemographic characteristics of patients, family members, and physicians (N=176).

Parameters		Patients (n=120)		Family members (n=24)		Physicians (n=32)	
		Included (n=67)	Excluded (n=53)	Included (n=16)	Excluded (n=8)	Included (n=25)	Excluded (n=7)
**Sex**							
	Male	11	18	2	1	13	3
	Female	56	35	14	7	12	4
**Age**							
	Average	51	50	46	49	42	49
	Maximum	85	87	62	62	69	56
	Minimum	17	17	23	33	28	29
**Civil status**							
	Married or cohabiting	43	37	8	7	-^a^	-
	Single	11	11	3	0	-	-
	Divorced	9	3	2	1	-	-
	Widowed	4	2	3	0	-	-
**Educational qualification**							
	Technical college or university degree	28	16	10	3	25	7
	Abitur	9	5	3	1	0	0
	Advanced technical college degree	6	5	0	1	0	0
	Secondary education	17	19	3	3	0	0
	Secondary modern school qualification	7	8	0	0	0	0
**Members of the household**							
	Average	2	5	3	3	-	-
	Maximum	5	2	5	5	-	-
	Minimum	0	0	0	0	-	-
**Age at diagnosis, years**							
	Average	37	37	4	15	-	-
	Maximum	74	79	37	47	-	-
	Minimum	0	0	0	0	-	-
**Disease severity**							
	No specification	0	0	1	0	-	-
	Low	6	3	0	0	-	-
	Medium	32	21	7	5	-	-
	Severe	28	29	8	3	-	-
**Profession**							
	Employed	27	25	16	5	25	7
	Unemployable	14	10	0	0	0	0
	Pensioner	20	14	0	2	0	0
	Student or scholar	1	2	0	0	0	0
	Homemaker	1	1	0	1	0	0
Special circumstances (further education or provision of work)		4	1	0	0	0	0
						
Medical rare disease experience		-	-	-	-	24	3

^a^The symbol indicates that data are not available.

**Table 3:** Ranking results of patients.

Parameters		Patients (n=67)				
		Local weight	SD	Global weight	Local ranking	Global ranking
**Medical issues**		.21	0.21		3 or 4	
	Diagnosis	.34	0.24	.070	2	6
	Therapy	.37	0.21	.076	1	5
	General disease pattern	.30	0.19	.062	3	9
**Research**		.21	0.17		3 or 4	
	Current studies	.32	0.22	.069	2	7
	Study results	.32	0.20	.068	3	8
	Registers	.36	0.26	.077	1	4
**Social help offers**		.30	0.19		1	
	Psychosocial counseling	.35	0.22	.103	1	1
	Self-help	.32	0.24	.095	3	3
	Sociolegal advice	.33	0.21	.098	2	2
Current events		.28	0.22		2	

**Table 4:** Ranking results of family members.

Parameters		Family members (n=16)				
		Local weight	SD	Global weight	Local ranking	Global ranking
**Medical issues**		.13	0.18		4	
	Diagnosis	.24	0.21	.031	2	8
	Therapy	.20	0.18	.025	3	9
	General disease pattern	.56	0.20	.071	1	3/4/5
**Research**		.22	0.20		2/3	
	Current studies	.31	0.21	.071	2	3/4/5
	Study results	.16	0.10	.037	3	7
	Registers	.52	0.23	.117	1	1
**Social help offers**		.22	0.16		2/3	
	Psychosocial counseling	.35	0.23	.075	1	2
	Self-help	.33	0.27	.071	2	3/4/5
	Sociolegal advice	.33	0.22	.070	3	6
Current events		.43	0.18	-	1	-

**Table 5:** Ranking results of physicians.

Parameters		Physicians (n=25)				
		Local weight	SD	Global weight	Local ranking	Global ranking
**Medical issues**		.13	0.17		4	
	Diagnosis	.23	0.16	.029	3	9
	Therapy	.37	0.17	.046	2	7
	General disease pattern	.40	0.19	.051	1	6
**Research**		.18	0.14		3	
	Current studies	.44	0.22	.078	1	3
	Study results	.25	0.18	.045	3	8
	Registers	.32	0.22	.057	2	5
**Social help offers**		.26	0.17		2	
	Psychosocial counseling	.29	0.11	.076	3	4
	Self-help	.32	0.20	.083	2	2
	Sociolegal advice	.40	0.20	.104	1	1
Current events		.42	0.17		1	

**Table 6:** Significance of differences between patients, family members, and physicians (n=108).

Parameters		Two-sample *t* test					
		Patients or families		Patients or physicians		Physicians or families	
		*t* statistic (degrees of freedom)	*P* value	*t* statistic (degrees of freedom)	*P* value	*t* statistic (degrees of freedom)	*P* value
**Medical issues**		1.60 (26)	.13	1.90 (55)	.06	0.04 (30)	.97
	Diagnosis	1.43 (26)	.17	2.59 (62)	.01	−0.45 (26)	.66
	Therapy	2.88 (26)	.01	0.07 (52)	.94	2.60 (31)	.01
	General disease pattern	−4.26 (22)	<.001	−2.50 (39)	.02	−1.85 (32)	.07
**Research**		−0.65 (21)	.52	0.59 (54)	.56	−0.98 (24)	.34
	Current studies	−0.26 (23)	.80	−1.98 (40)	.05	1.28 (34)	.21
	Study results	3.99 (46)	<.001	1.20 (46)	.21	1.98 (38)	.06
	Registers	−1.96 (25)	.06	0.87 (49)	.39	−2.44 (31)	.02
**Social help offers**		1.25 (27)	.28	0.19 (48)	.85	0.94 (34)	.35
	Psychosocial counseling	0.01 (22)	.99	2.05 (78)	.04	−1.13 (20)	.27
	Self-help	−0.12 (21)	.90	0.02 (48)	.98	−0.13 (26)	.90
	Sociolegal advice	0.13 (22)	.90	−1.50 (44)	.14	1.17 (30)	.25
Current events		−1.98 (26)	.06	−2.52 (54)	.01	0.10 (31)	.92

## Discussion

### Principal Findings

This study shows that rare diseases information categories are weighted very differently, resulting in subgroup specific preference weight structures, distributions, and ranking results. Although *medical issues* were rated as least important by all subgroups, none of the other information categories showed an overall group consensus.

Significant differences between subgroups were confirmed by *t* tests comparing subgroup specific local weights for the following comparisons: the priority weight of patients and family members in the categories *therapy*, *general disease pattern*, and *study results* differed significantly. Moreover, patients and physicians showed significant differences within the categories of *diagnosis*, *general disease pattern*, *current studies*, *psychosocial counseling*, and *current events*. Comparing physicians’ results against those of family members, *therapy* and *registers* showed statistical significance.

In quantifying these results, patients and family members showed diverging preference weights for 23% of the cases (3/13). On the other hand, patients and physicians showed different weights for 38% of the cases (5/13). Finally, physicians and family member’s weights diverged only in two cases (15%, 2/13). These results indicate that patients and physicians show a comparably high percentage of diverging opinions on the importance of health information, weakening our initial hypothesis that physicians initiate their search strategy based on the patient-physician interaction. These results should be discussed very carefully because the potential implications are hard to grasp. The statistical significance test was based on the local preference weight. However, the final result of the AHP was expressed as an absolute rank. Therefore, the results should be situated in the overall context. The local weights revealed significant differences in health information with regard to *therapy*. Specifically, patients put this category first (1) on the local level, whereas physicians put it last (3). Regarding the health information on *general disease patterns*, ranks were assigned inversely. Similar rank switches at the local level can be observed when comparing patients’ and physicians’ perspectives on information relating to *general disease patterns* and *psychological counseling*. Interestingly, *general disease patterns* were perceived as least important by patients (3), whereas physicians regarded it as most important (1). On the other hand, patients considered *psychosocial counseling* as the most important subcategory, whereas physicians considered it the least important.

Checking all subgroups for the sensitivity of results, a rank change could only be observed once. Therefore, we conclude that the results were relatively stable. These results are consistent with Danner et al [35], who interviewed patients while they were completing AHP questionnaires. Extreme values, which could lead to very unstable results, often go along with high inconsistencies. Per these findings, some extreme opinions could have been excluded because of the set CR threshold.

### Theoretical Contributions

Interestingly, all subgroups prioritized information on *social help offers* and *current events* over hard facts such as *medical issues* and *research*. This is perhaps because certain medical topics can be discussed directly with physicians following a diagnosis. Unfortunately, we cannot directly compare these findings with the findings of other studies, as the study participants, information categories, and indications vary greatly. However, patients receiving genomic results outlined that they preferred filtering information to avoid information overload and to avoid learning what their future might look like [36]. This anxiety about the future might explain why patients rated medical information as less important, despite the fact that it was named as a main search item in studies such as that of Morgan et al [31]. On the other hand, Anderson et al [37], as well as Schwarzer [38] reported consistent findings with Australian families suffering from genetic metabolic diseases and children with anorectal malformations, emphasizing the importance of self-help groups in the long run and psychosocial counseling when self-help reaches its limits. Dellve et al [39] also highlight the importance of psychosocial counseling for family members, especially parents with a child suffering from a rare disease. These findings also quantitatively support the importance of not only research networks, as advocated for by, for instance, Aymé and Schmidtke [40], but also social networks in the field of rare diseases and inclusion of these networks within national and international rare diseases information platforms, reflecting the unique importance of self-help initiatives in the field of rare diseases. Common diseases often do not need the support of self-help groups because research and political action have already been largely implemented. On the other hand, for rare diseases, many initiatives and knowledge extensions originate from these self-help groups [6]. However, patient initiatives continue to be put at the end of the line. Given that research- and patient-oriented websites still primarily offer either websites for physicians or for patients, even though information valuable to all stakeholders are presented, this makes cocreation and the exchange of opinions even more important.

The information category *registers* was the most important category for families (at rank 1); patients regarded it highly as well, ranking it in 4th place immediately after *social help offers*. Only physicians attributed a high relevance to *current studies*. This statement emphasizes the importance of providing information on rare diseases registers and appreciates the worldwide effort put into the development of such strategies [41], mirroring the importance of longitudinal data acquisition and analysis as numerous rare diseases are connected to a genetic predisposition [19]. These results emphasize the considerable involvement of family members, as they are potentially also affected.

Relatively little interest in study results can be explained through the communication of the results itself. Long et al [42] report that participants of studies receive results only in 33% of the cases. Only half of respondents saw an opportunity to even request the results. However, in this case, almost all respondents demanded researchers to at least sometimes offer the results. The strengthening of the communication of study results can be seen as an opportunity to improve the inclusion of health innovations in health care systems.

The present health information survey among physicians and senior patients reveals some major problems when comparing these results to those of other studies. Specifically, the results vary widely, especially because the health information categories were outlined differently [43]. This indicates that further subgroup analysis can be performed while controlling for influential factors such as age and indication. However, it should also be emphasized that our study forms the basis for an Internet platform for rare diseases and therefore focused on the major relevant stakeholders for this disease category.

Besides, research has often focused on topics such as information access [44] or barriers to information access [45], which leaves the question of how information needs are specified unanswered [46]. Further research is necessary to examine this topic in more detail. Nevertheless, the results have potential for further improving the basis of physician-patient communication.

### Practical Implications for Web-Based Health Information Provision

What do these results mean for rare diseases–related information providers such as ZIPSE? The differences between subgroups suggest that subgroup specific information is necessary. First, the ranking structure of rare diseases information categories can be translated, one-by-one, into website design by positioning topics in accordance with stakeholder priorities.

Besides, it seems advisable to considered Miller’s Law to avoid information overflow. It appreciated that the whole load of rare diseases Internet resources cannot be processed at once [47]. Limited perception capacities of human brains make it indispensable to only display the most important information at first glance. Miller’s Law states that the short-term memory of an average human brain can only absorb approximately 7 items at once, thus, limiting the effectiveness of Internet data processing. Moreover, considering Miller’s Law and potential information overflow, only the most important seven items should be included. Therefore, the findings suggest that information categories such as *general disease information* (9), *study results* (8), and *current studies* (7) do not need to be presented initially. In the case of a website especially designed for family members, *current events*, *registers*, *psychosocial counseling*, *self-help*, *sociolegal advice*, *current studies*, and *general disease pattern* should be presented first. On the other hand, physicians prioritized information on *current events*, *sociolegal advice*, *self-help*, *current studies*, *psychosocial counseling*, *registers*, and *general disease pattern*.

Nevertheless, another perspective should also be thought of at this point. From an educational point of view, this study also presents information categories that currently seem undervalued. For example, patients do not perceive *current studies* (7) or *study results* (8) as important, even though these results might hold crucial information for their disease treatment or maintenance. Family members do not perceive *diagnosis* (8) and *therapy* (9) as very valuable. Group representatives often advocate for their children or partners who are suffering from a rare disease to treat these information categories as more important. Moreover, even though approximately 60% of patients see physicians as the primary source of information [14], physicians do not perceive information on *diagnosis* (9), *therapy* (7), and *study results* (8) as important. Therefore, it seems advisable to discuss whether information should be located to improve its visibility and to reflect its importance for the major stakeholder, the patient. Consequently, whether physicians’ priorities should reflect patients’ interests as an *information lobbyist* also requires examination. First of all, it seems advisable to not only include the underlying results into the design of information platforms on rare diseases but also to discuss information placement with experts in the field and to fully disclose information placement strategies. However, we strive for a high involvement of patients, family members, and physicians to realize efficiency potentials for health care systems. This can only be accomplished by respecting the outcome of the decision-making process translating results one-to-one.

### Study Limitations

Data interpretation was a limitation. The AHP research sample size is still a topic of discussion. It has been highlighted that AHP does not require a particularly large sample size [48]. Other authors emphasized that there is no recommendation at all for AHP sample size [23]. Both sources base their statements on the fact that AHP reflects the opinion of the specific group and is thus a group decision-making tool. However, in this study, we raise preference weights, which should be representative for groups when an adequate sample size is achieved.

The quantitative aggregation technique shapes a clear-cut implementation structure for information categories. However, it must be acknowledged that the results illustrate the average opinions of rare diseases’ patients, physicians, and family members.

Another issue that should be recognized when interpreting study results is the exclusion of inconsistent answers as part of the AHP methodology. Dolan [49] found that of 20 patients, 90% were willing and capable of completing an AHP. Danner et al [34] argued that extreme values are often chosen to emphasize answers that are not willingly contributed to inconsistencies. In our study, patients delivered inconsistent answers 44% of the time, whereas family members and physicians did so in 34% and 22% of the cases, respectively. However, these results were excluded to follow theoretical AHP requirements.

During pretests of the questionnaire’s paper-based version, low consistency values were generated. Ranking cards were included as first choice assistive tools to mirror ranking results immediately. During interviews with patients and family members, this tool was very helpful and led to improved CR values. However, during interviewer-led AHPs, physicians refused to use it. Nevertheless, interviewers noted the shown ranking orders verbally. Finally, a ranking task was placed before each block of comparisons in the Web- and paper-based version.

Comparing physicians with patients, low participation rates are observed. VanGeest et al [50] stated that low participation rates are very common in physicians’ surveys. Postal and telephone approaches seem to be more effective than Web-based strategies. Monetary incentives were found to be an effective strategy to increase participation rates. Nonmonetary incentives reflected little changes. Unfortunately, no monetary funds were available for this study.

As already indicated, a change of medium was necessary. Initially, a paper-based version was implemented. After the first recruitment period, a Web-based questionnaire was also introduced to broaden the target group. Several studies such as those of Hirsch et al [51] and Coons et al [52] found differences between participation for paper-based and Web-based surveys. Therefore, it is beneficial to combine both approaches considering representativeness, thus capturing both infrequent and frequent Internet users.

Finally, sociodemographic data show a relatively large proportion of female participants. Literature and other rare diseases Internet providers disclaim that health information on rare diseases are more often searched for by women than by men. For instance, Morgan et al [13] determined that 95.7 % of all inquiries to the Genetic and Rare Disease Information Center came from women.

### Conclusions

This study describes an innovation in the involvement of patients, family members, and physicians in effectively gathering, structuring, and presenting health information in a world struggling with an information paradox, namely, health information overflow on the one hand and a major lack of information on rare conditions on the other. This innovation comes in the form of the chosen group decision-making tool, the AHP, which has helped transform individual qualitative perceptions into a measurable scale. Accordingly, the strength of our study is its transparent quantitative demonstration of the information needs of physicians, patients, and family members, which makes direct comparisons and simple implementation possible. More specifically, this study provides unique insights into the quantitative structure and distribution of information preferences, as well as the validity of results. We were able to verify significant differences between preference weights of patients, family members, and physicians for some items, suggesting that the importance of rare diseases information is perceived differently in these subgroups. User-oriented information providers should seek to address these differences and provide stakeholder-specific websites in accordance with the relevance of health information. Furthermore, the importance of social help offers and current events as part of the information package might be underpinned, with a particular emphasis on the importance of social networks in the field of rare diseases. The finding that communication of study results is potentially undervalued can be seen as an opportunity to improve the inclusion of information on health innovations in health care systems. As we strive for a high involvement of patients, family members, and physicians to realize efficiency potentials for health care systems, the relevance of health information should be directly translated. Results must not only be considered when creating national rare diseases information platforms such as the ZIPSE but also when updating, redesigning, and implementing national and international rare diseases information platforms.

However, as part of the cocreation process, we solely focused on the subgroups interested in information on rare diseases as an explanatory variable for different information needs. We suggest that future studies examine other potential explanatory variables such as for instance gender, educational background, and civil status.

Finally, our findings might be helpful for improving communication between patients, legal guardians or partners, and health advocates, who are closely intertwined. This seems to have high potential because social and professional networks often remain separate within discussions of rare diseases. Promoting a discussion between stakeholders can help in combining forces within the backdrop of a networking approach, which has already been communicated and pursued through the implementation of national rare diseases plans. An understanding network that engages in successful collaboration can improve the quality of life of those affected by rare diseases, as well as lessen the perceived disease burden.

## Appendix

Multimedia Appendix 1 Description of rare diseases information categories.

Multimedia Appendix 2 Questionnaire.

## Abbreviations

ACHSE: Allianz Chronisch Seltener Erkrankungen
AHP: analytic hierarchy process
CHERH: Center for Health Economics Research
CR: consistency ratio
NAMSE: Nationales Aktionsbündnis für Seltene Erkrankungen
SD: standard deviation
ZIPSE: Zentrales Informationsportal über seltene Erkrankungen
