# Supplementary material for: Shaping an Effective Health Information Website on Rare Diseases Using a Group Decision-Making Tool: Inclusion of the Perspectives of Patients, Their Family Members, and Physicians
Source: Interact J Med Res. 2017 Nov 20;6(2):e23. doi: 10.2196/ijmr.7352 (PMC5715203; doi:10.2196/ijmr.7352)
Supplement: Multimedia Appendix 1 [file ijmr_v6i2e23_app1.pdf]

Appendix 1. Description of rare diseases information categories

**Item**	**Description**
Medical issues	Information that contains medical background information about rare diseases (e.g., information about diagnostics, therapy, or disease pattern).
Diagnosis	Information about diagnostic procedures that a healthcare professional can identify as a rare disease and make a diagnosis. Additionally, contact information about specialized healthcare professionals or centers for rare diseases.
Therapy	Information on treatment procedures. Additionally, contact information about healthcare professionals that can treat people suffering from a rare disease.
General disease pattern	Information on reasons, symptoms, and progression of rare diseases.
Research	Information and results of efforts from scientists or pharmaceutical companies about new findings concerning rare diseases.
Current studies	Investigations about medical treatments of rare diseases that are scheduled or currently implemented and still searching for participants.
Study results	Results from current medical research.
Registries	Gathering of disease data in the long term to improve the treatment opportunities and to monitor the distribution of the diseases.
Social help offers	Contact data and information about counseling centers that can help people who are suffering from a rare disease.
Psychosocial counseling	Information and contact data that can provide people with psychosocial counseling concerning illness-related problems with family, friends, or at the work place.
Self-help	Contact information about groups of patients and close relatives to support each other.
Social-legal advice	Here you can find answers about questions dealing with services of statutory health insurance, labor law, or statutory pension funds.
Current events	Information and important appointments to public meetings where patients and affected persons can talk to healthcare staff.
