# Supplementary material for: Shaping an Effective Health Information Website on Rare Diseases Using a Group Decision-Making Tool: Inclusion of the Perspectives of Patients, Their Family Members, and Physicians
Source: Interact J Med Res. 2017 Nov 20;6(2):e23. doi: 10.2196/ijmr.7352 (PMC5715203; doi:10.2196/ijmr.7352)
Supplement: Multimedia Appendix 2 [file ijmr_v6i2e23_app2.pdf]

Informationsbedarf zu seltenen Erkrankungen

Seite 1

Zentrales Informationsportal für Seltene Erkrankungen
-Informationsnachfrage von Betroffenen, Angehörigen und Ärzten-

Gerne möchte wir Sie vorab zu den Hintergründen der folgenden Studie informieren.

Ziel des Forschungsprojektes ist der Aufbau eines zentralen Informationsportals über Seltene Erkrankungen (ZIPSE), welches sowohl Betroffenen und ihren Angehörigen als auch Ärzten, Therapeuten und Pflegekräften qualitätsgesicherte Informationen bereitstellt. Der Aufbau der Webseite ist ein vom Bundes Ministerium für Gesundheit gefördertes Projekt. Dabei sollen bereits verfügbare Informationen über seltene Erkrankungen auf der zentralen Portalseite gebündelt werden. Weiterführende Informationen zum Projekt finden Sie auf der Website der ZIPSE unter: www.portal-se.de.

Damit Sie in Zukunft genau die Informationen auf der ZIPSE-Website finden, die Sie besonders interessieren, möchten wir Sie bitten, an der folgenden Befragung teilzunehmen und uns Ihre persönliche Meinung mitzuteilen. Wir möchten von Ihnen erfahren, wie wichtig für Sie persönlich verschiedene Informationen sind. Bitte fragen Sie sich hierbei, welche Informationen zum aktuellen Zeitpunkt in Ihrer aktuellen Situation für Sie wichtiger sind. Dabei gibt es keine falschen Antworten! Zur Vorbereitung auf diese Befragung geben wir Ihnen auf den nächsten Seiten Erklärungen zu möglichen Bereichen, aus denen Sie Information zu seltenen Erkrankungen bekommen könnten. Bitte lesen Sie sich diese sorgfältig durch.

Für das Ausfüllen des Fragebogens benötigen Sie circa 10 Minuten.
Vielen Dank, dass Sie sich die Zeit nehmen an unserer Studie teilzunehmen!

Seite 2

Ihre Daten werden selbstverständlich vertraulich behandelt und ausschließlich im Rahmen des ZIPSE Projektes verwendet. Um dies zu gewährleisten, werden Ihre Angaben pseudonymisiert abgespeichert. Die Zugangsberechtigung zu den erhobenen Daten liegt ausschließlich bei den Mitarbeitern des Projektes. Es werden keine Daten an Dritte weitergegeben.
Es entsteht Ihnen kein Nachteil, falls Sie nicht an der Befragung teilnehmen möchten.

Ich bin damit einverstanden, dass die erhobenen Daten im Rahmen des ZIPSE Projektes verwendet werden. *

Ja

Seite 3

Bitte lesen Sie nun sorgfältig die Beschreibungen der einzelnen Bereiche und Arten von möglichen Informationen für die ZIPSE-Website durch, die für die ZIPSE Webseite in Frage kommen können. Entscheiden Sie dann im Anschluss welche Informationen Ihnen ganz persönlich wichtig sind.

----

1. Medizinische Fragen
Unter diesem Bereich verstehen wir Informationen, die etwas mit dem medizinischen Hintergrund einer seltenen Erkrankung zu tun haben. Darunter fallen zum Beispiel Fragen zur Diagnose, der Therapie oder der Entstehung von einer seltenen Erkrankung.

a) Diagnosefindung:
§ Leistungserbringer: Namen, Adressen, Telefonnummern, Homepages von Ärzten, die auf die Diagnose von seltenen Erkrankungen spezialisiert sind.
§ Diagnoseverfahren: Methoden in der Medizin und ihr Ablauf, mit denen seltene Erkrankungen erkannt werden können.

b) Therapie:
§ Behandelnde Leistungserbringer: Namen, Adressen, Telefonnummern, Homepages von Ärzten und Therapeuten, die auf die Behandlung (Therapie) von seltenen Erkrankungen spezialisiert sind.
§ Behandlungsverfahren: Informationen, die Behandlungsmethoden von seltenen Krankheiten beschreiben (z.B. Operationen oder Medikamente).

c) Allgemeines zum Krankheitsbild:
§ Ursachen: Hier werden die Auslöser von seltenen Erkrankungen erklärt, soweit diese bekannt sind. Zum Beispiel können genetische Defekte die Ursache sein.
§ Häufigkeiten: Unter diesem Punkt ist zu finden, wie viele Personen von der seltenen Erkrankung betroffen sind.
§ Symptome: An dieser Stelle werden Informationen gegeben, die Symptome oder typische Erscheinungen der Krankheit beschreiben.
§ Verlauf: Das bedeutet, welche Veränderungen sich bei den Patienten mit der Zeitergeben und in welcher Phase der Krankheit Veränderungen stattfinden.

----

2. Forschung
Der zweite große Bereich stellt Informationen zur Forschung bereit. Das bedeutet, dass es hier unter anderem Informationen dazu gibt, welche Bemühungen Arzneimittelhersteller gerade tätigen oder was Wissenschaftler Neues über seltene Erkrankungen herausfinden konnten.

a) Anlaufende Studien:
Untersuchungen von seltenen Erkrankungen, die in Planung sind bzw. gerade anlaufen und noch nach Studienteilnehmern suchen.

b) Studienergebnisse:
Hierunter werden Ergebnisse aus der aktuellen medizinischen Forschung verstanden.

c) Register:
Hier werden Krankheitsdaten langfristig gesammelt. Das Ziel ist es, Behandlungsmöglichkeiten weiterzuentwickeln und die Verteilung der Krankheit zu beobachten. Außerdem steht hier, wie man sich als Patient/in registrieren lassen kann.

----

3. Aktuelle Veranstaltungen
Hierunter fallen gemeinsame Maßnahmen von Patienten und Gesundheitspersonal, um Informationen auszutauschen oder die Erkrankung ins Bewusstsein der Öffentlichkeit zu rücken (z.B.: Termine von Aktionstagen wie dem Tag der Seltenen Erkrankungen).

----

4. Soziale Beratungs- und Hilfsangebote
Hiermit sind Kontaktdaten und Informationen zu Beratungsstellen gemeint, die Menschen mit seltenen Erkrankungen weiterhelfen können. Darunter fallen ganz unterschiedliche Bereiche, wie Hilfe zu Anträgen und Rechtsansprüchen, aber auch Informationen und Beratung zu psychischen Belastungen und der Selbsthilfe.

a) Sozialrechtliche Beratung:
Hier werden Fragen zu Krankenkassenleistungen, Arbeitsrecht, Rentenversicherung und andere beantwortet. Man kann sich hier über alle möglichen Anträge und Erstattungsfragen informieren.

b) Psychosoziale Beratung:
Hier finden Sie Informationen und Kontakte zu Beratungsangeboten bei erkrankungsbedingten Problemen innerhalb der Familie, im Freundeskreis oder am Arbeitsplatz zeigen.

c) Selbsthilfe:
§ Persönliche Kontakte: Hier gibt es Kontaktinformationen zu Selbsthilfegruppen, die sich in Ihrer Umgebung treffen und an denen teilgenommen werden kann.
§ Online Kontakte: An dieser Stelle finden Sie Verweise zu Homepages, Foren im Internet und E-Mailadressen, über die sich Betroffene austauschen können. Es gibt Erfahrungsberichte von anderen Menschen mit seltenen Erkrankungen oder Angehörigen.

Bitte bestätigen Sie kurz, dass Sie die Beschreibung der Informationskategorien gelesen und verstanden haben. *

- ja
- nein

Seite 4

Bitte nehmen Sie sich nun Zeit folgende Fragen aus Ihrer ganz persönlichen Sicht zu beantworten. Bitte fragen Sie sich hierbei, welche Informationen zum aktuellen Zeitpunkt in Ihrer aktuellen Situation für Sie wichtiger sind. Es gibt keine falschen Antworten!

Seite 5

Bitte überlegen Sie sich zunächst, welche der Informationen der Wichtigkeit nach für Sie an erster, zweiter, dritter und vierter Stelle stehen und tragen Sie dann die Reihenfolge ein. *

An erster Stelle steht, die für Sie persönlich wichtigste Information und an letzter Stelle steht, die für Sie persönlich am wenigsten wichtige Information.

- Medizinische Fragen
- Forschung
- Aktuelle Veranstaltungen
- Soziale Beratungs- und Hilfsangebote

Tragen Sie anschließend ein wie viel die Informationen für Sie wichtiger sind.

Wie wichtig sind Ihnen Informationen zu Medizinischen Fragen im Vergleich zu Informationen zur Forschung? *

	9 absolut wichtiger	7 sehr viel wichtiger	5 erheblich wichtiger	3 etwas wichtiger	-1- gleich wichtig	3 etwas wichtiger	5 erheblich wichtiger	7 sehr viel wichtiger	9 absolut wichtiger	
Medizinische Fragen										Forschung

Wie wichtig sind Ihnen Informationen zu Medizinischen Fragen im Vergleich zu Informationen zu Aktuellen Veranstaltungen? *

	9	7	5	3	-1-	3	5	7	9	
Medizinische Fragen										Aktuellen Veranstaltungen

Wie wichtig sind Ihnen Informationen zu Medizinischen Fragen im Vergleich zu Sozialen Beratungs- und Hilfsangeboten? *

	9	7	5	3	-1-	3	5	7	9	
Medizinische Fragen	◯ ◯	◯ ◯	◯ ◯	◯ ◯	◯	◯ ◯	◯ ◯	◯ ◯	◯ ◯	Soziale Beratungs- und Hilfsangeboten

Wie wichtig sind Ihnen Informationen zur Forschung im Vergleich zu Informationen über Aktuelle Veranstaltungen? *

	9	7	5	3	-1-	3	5	7	9	
Forschung	◯ ◯	◯ ◯	◯ ◯	◯ ◯	◯	◯ ◯	◯ ◯	◯ ◯	◯ ◯	Aktuelle Veranstaltungen

Wie wichtig sind Ihnen Informationen zur Forschung im Vergleich zu Informationen über Soziale Beratungs- und Hilfsangebote? *

	9	7	5	3	-1-	3	5	7	9	
Forschung	◯ ◯	◯ ◯	◯ ◯	◯ ◯	◯	◯ ◯	◯ ◯	◯ ◯	◯ ◯	Soziale Beratungs- und Hilfsangebote

Wie wichtig sind Ihnen Informationen zu Aktuellen Veranstaltungen im Vergleich zu Informationen über Soziale Beratungs- und Hilfsangebote? *

	9	7	5	3	-1-	3	5	7	9	
Aktuelle Veranstaltungen	◯ ◯	◯ ◯	◯ ◯	◯ ◯	◯	◯ ◯	◯ ◯	◯ ◯	◯ ◯	Soziale Beratungs- und Hilfsangebote

Seite 6

Bitte überlegen Sie sich zunächst, welche der Informationen der Wichtigkeit nach für Sie an erster, zweiter und dritter Stelle stehen und tragen Sie dann die Reihenfolge ein. *

An erster Stelle steht, die für Sie persönlich wichtigste Information und an letzter Stelle steht, die für Sie persönlich am wenigsten wichtige Information.

- [▾] Diagnose
- [▾] Therapie
- [▾] Allgemeines zum Krankheitsbild

Tragen Sie anschließend ein wie viel die Informationen für Sie wichtiger sind.

Wie wichtig sind Ihnen Informationen zur Diagnosefindung im Vergleich zu Informationen über zur Therapie? *

	9<br>absolut<br>wichtiger	7<br>sehr viel<br>wichtiger	5<br>erheblich<br>wichtiger	3<br>etwas<br>wichtiger	-1-<br>gleich<br>wichtig	3<br>etwas<br>wichtiger	5<br>erheblich<br>wichtiger	7<br>sehr viel<br>wichtiger	9<br>absolut<br>wichtiger	
Diagnose-<br>findung										Therapie

Wie wichtig sind Ihnen Informationen zur Diagnosefindung im Vergleich zu Informationen zum Krankheitsbild? *

	9	7	5	3	-1-	3	5	7	9	
Diagnosefindung										Allgemeines zum Krankheitsbild

Wie wichtig sind Ihnen Informationen zur Therapie im Vergleich zu Informationen zum Krankheitsbild? *

	9	7	5	3	-1-	3	5	7	9	
Therapie										Allgemeines zum Krankheitsbild

Seite 7

Bitte überlegen Sie sich zunächst, welche der Informationen der Wichtigkeit nach für Sie an erster, zweiter und dritter Stelle stehen und tragen Sie dann die Reihenfolge ein. *

An erster Stelle steht, die für Sie persönlich wichtigste Information und an letzter Stelle steht, die für Sie persönlich am wenigsten wichtige Information.

- Anlaufenden Studien
- Register
- Studienergebnisse

Tragen Sie anschließend ein wie viel die Informationen für Sie wichtiger sind.

Wie wichtig sind Ihnen Informationen zu Anlaufenden Studien im Vergleich zu Informationen zu Studienergebnissen? *

	9<br>absolut<br>wichtiger	7<br>sehr viel<br>wichtiger	5<br>erheblich<br>wichtier	3<br>etwas<br>wichtiger	-1-<br>gleich<br>wichtig	3<br>etwas<br>wichtiger	5<br>erheblich<br>wichtiger	7<br>sehr viel<br>wichtiger	9<br>absolut<br>wichtiger	
Anlaufende<br>Studien										Studienergebnisse

Wie wichtig sind Ihnen Informationen zu Anlaufenden Studien im Vergleich zu Informationen über Register?

	9	7	5	3	-1-	3	5	7	9	
Anlaufende Studien	○ ○	○ ○	○ ○	○ ○	○	○ ○	○ ○	○ ○	○ ○	Register

Wie wichtig sind Ihnen Informationen zu Studienergebnissen im Vergleich zu Informationen über Register? *

	9	7	5	3	-1-	3	5	7	9	
Studienergebnisse	○ ○	○ ○	○ ○	○ ○	○	○ ○	○ ○	○ ○	○ ○	Register

Seite 8

Bitte überlegen Sie sich zunächst, welche der Informationen der Wichtigkeit nach für Sie an erster, zweiter und dritter Stelle stehen und tragen Sie dann die Reihenfolge ein. *

An erster Stelle steht, die für Sie persönlich wichtigste Information und an letzter Stelle steht, die für Sie persönlich am wenigsten wichtige Information.

- Sozialrechtliche Beratung
- Psychosoziale Beratung
- Selbsthilfe

Tragen Sie anschließend ein wie viel die Informationen für Sie wichtiger sind.

Wie wichtig sind Ihnen Informationen zur Sozialrechtlichen Beratung im Vergleich zu Informationen über Psychosoziale Beratung? *

	9 absolut wichtiger	7 sehr viel wichtiger	5 erheblich wichtiger	3 etwas wichtiger	-1- gleich wichtig	3 etwas wichtiger	5 erheblich wichtiger	7 sehr viel wichtiger	9 absolut wichtiger	
Sozialrechtliche Beratung	○ ○	○ ○	○ ○	○ ○	○	○ ○	○ ○	○ ○	○ ○	Psychosoziale Beratung

Wie wichtig sind Ihnen Informationen zur Sozialrechtlichen Beratung im Vergleich zu Informationen über Selbsthilfe? *

	9	7	5	3	-1-	3	5	7	9	
Sozialrechtliche Beratung	○ ○	○ ○	○ ○	○ ○	○	○ ○	○ ○	○ ○	○ ○	Selbsthilfe

Wie wichtig sind Ihnen Informationen zur Psychosozialen Beratung im Vergleich zu Informationen über Selbsthilfe? *

	9		7		5		3		-1-		3		5		7		9	
Psychosoziale Beratung	○	○	○	○	○	○	○	○	○	○	○	○	○	○	○	○	○	Selbsthilfe

Seite 9

Bitte beantworten Sie abschließend noch ein paar Fragen zu Ihrer Person.

Alter *

[ ]

Geschlecht *

○ männlich

○ weiblich

Sie sind ... *

○ Betroffene(r) einer seltenen Erkrankung

○ Arzt/Ärztin

○ Familienangehörige(r)

○ [ ]

Falls Sie Arzt oder Ärztin sind, welcher Fachrichtung gehören Sie an?

[ ]

Mit welcher seltenen Erkrankung(en) haben oder hatten Sie zu tun? *

Falls Sie bedenken haben, dass die Nennung des Krankheitsnamens Rückschlüsse auf Ihre Person zulässt, können Sie an dieser Stelle auch gerne einen Überbegriff angeben.

Sie sind ... *

- ◯ ledig
- ◯ getrennt
- ◯ geschieden
- ◯ verheiratet oder zusammenlebend
- ◯ verwitwet

Ihr höchster Bildungsabschluss ist ... *

- ◯ Berufsreife (Hauptschule)
- ◯ Abitur
- ◯ Mittlere Reife (Realschule)
- ◯ Fachhochschule/Universität
- ◯ Fachhochschulreife
- ◯ Kein Bildungsabschluss

Berufstätigkeit *

◯ Erwerbs- bzw. berufstätig

◯ Student-in/Schüler-in

◯ Arbeitssuchend

◯ Hausfrau/-mann

◯ Vorübergehend beurlaubt

◯ Rentner/Pensionär

◯ Arbeitsunfähigkeit

◯ Spezielle Situation (Weiterbildung/Arbeitsbeschaffung)

Bitte beantworten Sie folgende Fragen falls Sie Angehörige(r) oder Betroffene(r) sind.

In welchem Alter wurde die Krankheit diagnostiziert?

[ ]

Wie viele Personen (inkl. der eigenen Person) leben in Ihrem Haushalt?

[ ]

Wie empfinden Sie die Schwere Ihrer/der Krankheit?

◯ leicht

◯ mittel

◯ schwer

Seite 10

Wir möchten uns ganz herzlich für Ihre Zeit und Mühe bedanken!

Falls Sie weitere Informationen zu den Hintergründen der Studie benötigen, besuchen Sie gerne unsere Projektwebseite unter: www.portal-se.de .

» Umleitung auf Schlussseite von Umfrage Online
